# Reinforced Palmprint Reconstruction Attacks in Biometric Systems

**DOI:** 10.3390/s22020591

**Published:** 2022-01-13

**Authors:** Yue Sun, Lu Leng, Zhe Jin, Byung-Gyu Kim

**Affiliations:** 1 Key Laboratory of Jiangxi Province for Image Processing and Pattern Recognition, Nanchang Hangkong University, Nanchang 330063, China; 1916085212108@stu.nchu.edu.cn (Y.S.); jin.zhe@gmail.com (Z.J.); 2 School of Artificial Intelligence, Anhui University, Hefei 230039, China; 3 Department of IT Engineering, Sookmyung Women’s University, Seoul 04310, Korea

**Keywords:** reinforced biometric reconstruction attack, palmprint recognition, modification constraint within neighborhood, batch member selection, visual quality, naturalness

## Abstract

Biometric signals can be acquired with different sensors and recognized in secure identity management systems. However, it is vulnerable to various attacks that compromise the security management in many applications, such as industrial IoT. In a real-world scenario, the target template stored in the database of a biometric system can possibly be leaked, and then used to reconstruct a fake image to fool the biometric system. As such, many reconstruction attacks have been proposed, yet unsatisfactory naturalness, poor visual quality or incompleteness remains as major limitations. Thus, two reinforced palmprint reconstruction attacks are proposed. Any palmprint image, which can be easily obtained, is used as the initial image, and the region of interest is iteratively modified with deep reinforcement strategies to reduce the matching distance. In the first attack, Modification Constraint within Neighborhood (MCwN) limits the modification extent and suppresses the reckless modification. In the second attack, Batch Member Selection (BMS) selects the significant pixels (SPs) to compose the batch, which are simultaneously modified to a slighter extent to reduce the matching number and the visual-quality degradation. The two reinforced attacks can satisfy all the requirements, which cannot be simultaneously satisfied by the existing attacks. The thorough experiments demonstrate that the two attacks have a highly successful attack rate for palmprint systems based on the most state-of-the-art coding-based methods.

## 1. Introduction

**Figure 1:**
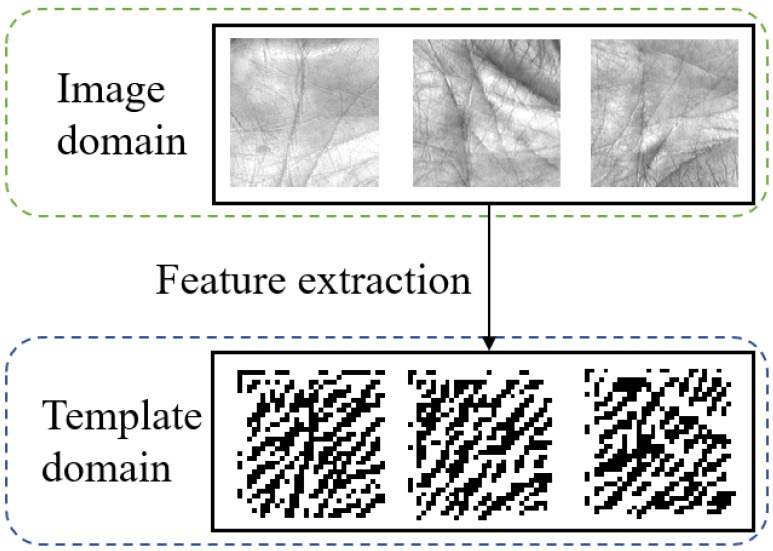
The two domains in biometric systems.

In secure identity management systems, biometric signals can be acquired with different sensors and recognized for automatic authentication/identification [1]. Generally, biometric recognition includes signal acquisition, pre-processing, feature extraction, and matching. The acquired signals are usually images for many biometric modalities, such as the face, iris, and palmprint. Feature extraction generates the templates that are transmitted to the communication channels and stored in databases. The probe template and gallery template are compared for the recognition decision. To reduce the privacy leakage from the original images, and also reduce the transmission burden and storage load, original biometric images are typically neither transmitted nor stored. As shown in Figure 1, images are in the image domain, while templates are in the template domain.

Unfortunately, biometric systems suffer from various attacks [2,3,4], which compromise their security management in many applications, such as industrial IoT. Reconstruction attacks have not been sufficiently considered, but they seriously threaten the security of identity management systems. In reconstruction attacks, an internal attacker can obtain the target template of a genuine user, which is stored in the database. Then the attacker uses the target template to reconstruct the corresponding fake original image to impersonate the target/genuine user and cheat the biometric system.

The basic requirement of a reconstruction attack is “**similarity**”. To satisfy similarity, the dissimilarity (/similarity) between the templates of the reconstructed fake image and target template of the genuine user is less (/higher) than a threshold, and then the attack is successful.

However, similarity, which is measured in the template domain, is not enough for a state-of-the-art reconstruction attack. To further comprehensively evaluate reconstruction attacks, three requirements in the image domain are defined as follows.

**Naturalness**: The reconstructed images should look like biometric images.

**Visual quality**: The reconstructed images should not have a remarkable noise-like appearance that implies they are fake images.

**Completeness**: The whole original image, rather than the region of interest (ROI), should be reconstructed. Since the acquired images are firstly pre-processed, such as segmentation and ROI localization, the original complete biometric image must be reconstructed.

It is highly difficult to simultaneously satisfy all the aforementioned indicators; thus, it is necessary to employ some reinforcement strategies to improve the attack performance.

Palmprint is a promising and representative biometric modality; that is, the methods for palmprint recognition can be conveniently transplanted or extended to other biometric modalities, so the reconstruction attacks in this paper are conducted on palmprint systems [5].

**Figure 2:**
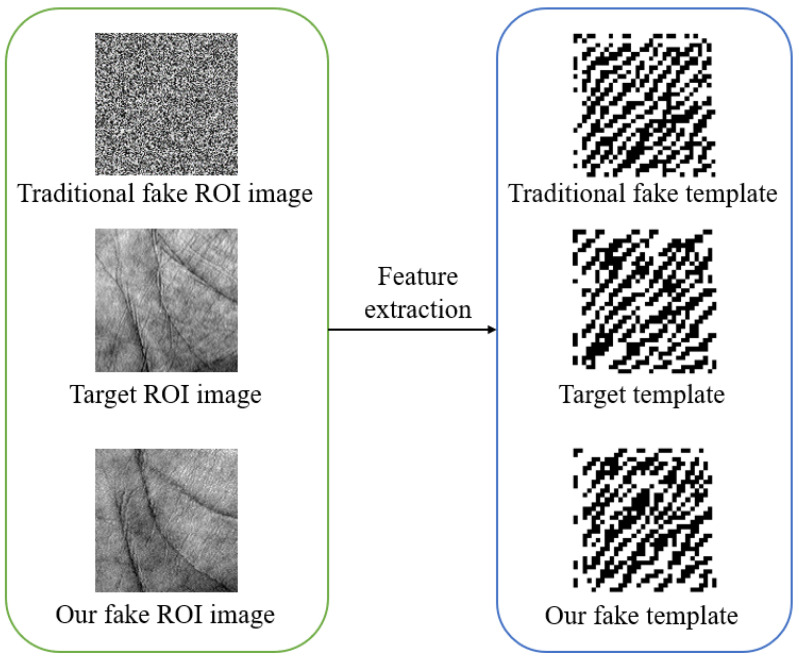
The images and templates in the reconstruction attack.

As shown in Figure 2, the traditional and our fake ROI images were reconstructed from the hill-climbing (HC) algorithm and our method, respectively. Both the traditional and our fake ROI images satisfy similarity; however, traditional fake ROI images have neither high naturalness nor high visual quality. Our method can reconstruct complete fake palmprint images with high naturalness and high visual quality.

This paper develops two novel reinforced palmprint reconstruction attacks based on reinforcement strategies, and the main contributions are summarized as follows:

(1) In order to comprehensively evaluate biometric reconstruction attacks in identity management systems, more indicators are proposed, including similarity, naturalness, visual quality, and completeness.
(2) Any palmprint, which can be easily obtained, is used as the initial image, so naturalness can be ensured. The ROI of the initial image is iteratively modified with deep reinforcement strategies to reduce the matching distance. There is no remarkable sudden change near the boundaries of ROI in the complete fake image, so both visual quality and completeness can be satisfied.
(3) In the first attack, Modification Constraint within Neighborhood (MCwN) is proposed to limit the modification extent and suppress the reckless modification to enhance the naturalness and visual quality.
(4) In the second attack, Batch Member Selection (BMS) is proposed to select the significant pixels (SPs) to compose the batch, in which the SPs, i.e., the batch members, are modified simultaneously to reduce the matching number, i.e., computational complexity. Since the pixels in the batch are modified together, their modifications are slighter, and accordingly both the naturalness and visual quality are maximized.

The experiments were sufficient and confirm that two reinforced palmprint reconstruction attacks have a highly successful attack rate for the palmprint systems based on the most state-of-the-art coding-based methods. In addition, the two reinforced attacks can satisfactorily meet all the indicators, which cannot be simultaneously satisfied by the existing attacks.

The rest of this paper is organized as follows. Section 2 introduces the related works on biometric reconstruction attacks and palmprint recognition methods. Section 3 specifies the proposed novel palmprint reconstruction attacks. The experiments are discussed in Section 4. Finally, the conclusions are drawn in Section 5.

## 2. Related Works

### 2.1. Reconstruction Attacks

Some reconstruction attacks have been developed and conducted on some biometric modalities, including fingerprint, face, iris, and palmprint. Table 1 compares the reconstruction attacks. Similarity is the basic requirement, so each attack must satisfy it. Similarity requirement is typically measured by a successful attack rate. The existing methods cannot satisfy the four evaluation indicators simultaneously, including similarity, naturalness, visual quality, and completeness.

Compared with other biometric modalities, it is difficult to reconstruct complete palmprint images. The reconstructed face ROI can be directly pasted onto a complete face image due to the smoothness of the face skin. The inner hole and outside region of the iris are the pupil and the eyelids/sclera, respectively. The inner and outer boundaries of the iris are sharp, so it is easy to paste the reconstructed iris ROI onto a complete iris image. A palmprint ROI is typically a region on the palm. Visual quality requires the boundaries of the ROI to be smooth. If the reconstructed palmprint ROI is directly pasted onto a complete palmprint image, the boundaries of the ROI are inevitably sharp. A complete palmprint image is used as the initial image in our methods, which is slightly modified, so the degradation of the visual quality is slight.

Wang et al.’s method [27] trained a generative adversarial network (GAN) with a large number of palmprint images and used the trained generator to generate a large number of palmprint images to attack the palmprint recognition system by brute force. Although the palmprint images generated by Wang et al.’s method [27] have good naturalness and visual quality, a large number of palmprint images are required to train the GAN, which is also time-consuming. In contrast, only one palmprint is required in the attacks in this paper, and any palmprint image, which is easily obtained, can be used as this required image.

### 2.2. Palmprint Recognition

A palmprint is a promising and representative biometric modality. Palmprint recognition methods can be roughly categorized into subspace-based [28,29], statistical-based [30,31], deep-learning-based [32], and coding-based [33] methods. Coding-based methods are free from training and have a low storage cost and fast computational speed, so they are popular for palmprint recognition.

Texture is one of the most discriminative features in palmprint images, so many existing palmprint recognition methods extract the discriminative texture features and coded them according to pre-defined rules [34]. The main problems for coding-based methods include how to accurately describe the texture and exactly extract the discriminative features [35].

Zhang et al. [36] proposed PalmCode, which utilized a 2D Gabor filter along “45°” to extract the palmprint feature. PalmCode only extracts single-orientation texture while ignoring the texture information along other orientations. To relieve this problem, Guo et al. [37] proposed Binary Orientation Co-occurrence Vector (BOCV), which used six Gabor filters along different orientations to extract the texture features, where the final matching distance was the average value of the six matching distances. Sun et al. [38] proposed Ordinal Code, where the ordinal information was extracted and coded as the feature from three pairs of the orthogonal orientations. Kong et al. [39] proposed Fusion Code, which applied four Gabor filters to extract the texture and phase as the features.

Dominant orientation is another popular feature for palmprint recognition. Kong and Zhang [40] proposed Competitive Code (CompCode), which used six Gabor filters to extract features, and then selected the index of the best response at each position as the feature. Similar to CompCode, Jia et al. [41] proposed Robust Line Orientation Code (RLOC). The difference between RLOC and CompCode was the choice of the filter; a modified finite Radon transform, rather than Gabor filter, was used in RLOC to extract the texture features. Fei et al. [42] proposed a Double Orientation Code (DOC) with the fine-tuned Gabor filters to describe each pixel more accurately. DOC selected the indices of the top-2 best responses at each position as the feature. Xu et al. [43] proposed Discriminative Competitive Code (DCC) and Discriminative Robust Competitive Code (DRCC). The dominant orientation and the relationship between its two neighbor orientations were coded as the feature. Different from DCC, DRCC applied a Gaussian filter to smooth the palmprint image.

## 3. Methodology

Two novel reinforced palmprint reconstruction attacks with reinforcement strategies, namely, Modification Constraint within Neighborhood (MCwN) and Batch Member Selection (BMS), are proposed in this paper, which can satisfy the four indicators simultaneously.

### 3.1. Modification Constraint within Neighborhood

Hill-climbing (HC) method is a simple greedy search algorithm that selects a top-like solution from the proximity to the current solution until it reaches a locally optimal solution. In HC, all pixels are modified to reduce the matching distance until the reconstructed image can satisfy similarity. Unfortunately, the optimization objective of ***similarity*** cannot maintain, and even damages, the ***naturalness*** and ***visual quality***.

Any palmprint, which can be easily obtained, can be used as the initial image in this paper, so the naturalness is satisfied. Strong modification can reduce the matching distance (improve similarity), but definitely damages the naturalness and visual quality. The slighter the modification is, the higher the naturalness and visual quality are. Modification Constraint within Neighborhood (MCwN) is proposed for “single-pixel modification” to reduce the modification extent, and accordingly maximize the naturalness and visual quality; so, MCwN satisfactorily balances the conflict between similarity and naturalness/visual quality.

The modification includes direction and stride. There are two modification directions—positive and negative directions—that control the increase and decrease in pixel value, respectively. Stride is the modification range every time.

f denotes the current version of the palmprint ROI image with M×N size, and $f^{\prime }$ is its modified version. f(x,y) is the pixel in f, 1≤x≤M, 1≤y≤N. Δf and k represent the modification direction and stride, respectively. Then the modified version is

(1) $$f^{\prime }\left(x,\;y\right)=\Delta f\times k+f\left(x,y\right)$$

Δf=+1 and −1 for positive and negative directions, respectively. The set of neighbors at the current location is represented by $f\left(s_{x},s_{y}\right)$.

(2) $$s_{x}=\left\{x-1,x,x+1\right\}$$

(3) $$s_{y}=\left\{y-1,y,y+1\right\}$$

(4) $$R_{max}=\max\left(\{f(s_{x},s_{y}\right)\})$$

(5) $$R_{min}=\min\left(\{f(s_{x},s_{y}\right)\})$$

where $R_{max}$ and $R_{min}$ represent the maximum and minimum pixel values in the neighborhood, respectively. The normalized distance $dis^{\prime }$ is measured by the dissimilarity between the template of $f^{\prime }\left(x,\;y\right)$ and the target template in the database. The normalized distance dis is measured by the dissimilarity between the template of f(x,y) and target template.

For each pixel, firstly, Δf=−1. If the two conditions are both satisfied, namely $f^{\prime }\left(x,\;y\right)\in \left[R_{min},\;R_{max}\right]$ and $dis^{\prime }\leq dis$, $f\left(x,y\right)=f^{\prime }\left(x,\;y\right)$ is implemented; that is, the pixel value is changed from f(x,y) to $f^{\prime }\left(x,\;y\right)$, and then the modification position moves to the next pixel.

If the two conditions are not both satisfied, Δf=+1. Then, if the two conditions are both satisfied, $f=f^{\prime }$ is implemented; that is, the pixel value is changed from f(x,y) to $f^{\prime }\left(x,\;y\right)$, and then the modification position moves to the next pixel.

If the current pixel is not modified, the modification position directly moves to the next pixel.

Please note that $dis^{\prime }\leq dis$ is one condition. The performance of $dis^{\prime }<dis$ is not good because the modification is not conducted if $dis^{\prime }=dis$. In contrast, in the condition $dis^{\prime }\leq dis$, the modifications $dis^{\prime }=dis$ are conducted, which ensure the momentum is enough to persistently keep reducing the matching distance. One experiment in Section 4.3 confirms this conclusion.

The traversal way is from left to right, and from top to bottom. The above single-pixel modification is conducted on each pixel until $dis^{\prime }$ is less than the threshold or the maximum number of matchings (iterations) is reached.

### 3.2. Batch Member Selection

The effect of each single-pixel modification on similarity is slight. The interference probably exists between the single-pixel modifications of the adjacent pixels; i.e., the latter modification probably damages the effects of the former modifications. Thus, single-pixel modification is time-consuming and not highly effective. In addition, single-pixel modification easily damages the smoothness while enhancing the sharpness, so naturalness and visual quality are probably degraded.

In ***batch modification***, all the pixels in the batch are modified together, so its effect on similarity is more remarkable than that of single-pixel modification. Thus, batch modification can sharply reduce the matching distance (improve similarity) and the number of matching (iteration). Furthermore, batch modification avoids the degradation of naturalness and visual quality. Batch Member Selection (BMS) is critical in batch modification, i.e., how to select the significant pixels as the members of the batch. The SPs, i.e., batch members, are modified simultaneously. The SPs are defined as the pixels that have more remarkable effects on similarity. Only the SPs are modified while the other pixels are unchanged, which maximizes the naturalness and visual quality.

In the existing coding-based palmprint recognition methods, the upper-left pixel of each 4 × 4 block is typically selected as the downsampled representative of this block. Thus, the upper-left pixels of the blocks are more important. However, directly modifying the upper-left pixels definitely damages the naturalness and visual quality. To solve this problem, it is necessary to select the SPs to compose the modification batch and conduct batch modification.

The pixels with large absolute values in a filter typically have great effects on similarity, so they are selected as the SPs. The real value of an entry in the filter is α, and |·| is the absolute value function. The absolute values of the entries in the filter is |α|. |α| is normalized to ${\left|\alpha \right|}_{N}$ in the range [0, 1].

(6) $${\left|\alpha \right|}_{N}=\frac{\left|\alpha \right|}{{\left|\alpha \right|}_{max}-{\left|\alpha \right|}_{min}}$$

${\left|\alpha \right|}_{max}$ and ${\left|\alpha \right|}_{min}$ are the maximum and minimum |α|. A threshold value τ is used to control the number of SP. If |α|≥τ, the pixel is selected as SP. The larger τ is, the smaller the number of SP is.

SPs are divided into two sets: positive SP if α ≥ 0, and negative SP if α < 0. To enhance the response, the values of positive SPs are increased, i.e., Δf=+1; in turn, the values of negative SPs are decreased, i.e., Δf=−1.

If $dis^{\prime }\leq dis$, batch modification is conducted, and then the modification position moves to the next batch. The traversal way is from left to right, and from top to bottom. The above single-pixel modification is conducted on each pixel until $dis^{\prime }$ is less than the threshold or the maximum number of matchings (iterations) is reached.

Some palmprint recognition systems have multiple filters, each filter producing a template. The filters have their specific modification batches, and batch modification is conducted on these filters in turn.

**Figure 3:**
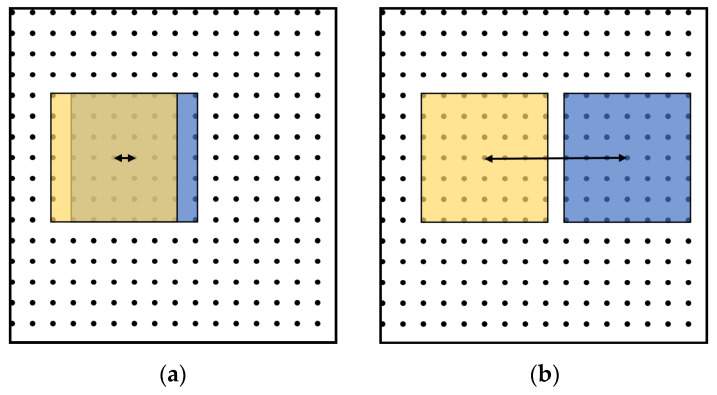
Two windows of the filter for BMS: (**a**) overlapping; (**b**) traversal gap.

The window of the filter for BMS slides from left to right, and from top to bottom. The SPs in each window are selected and compose the batch in this window. The batch modifications in two overlapped windows probably have conflicts; that is, the latter modification probably damages the effects of the former modifications. To avoid this problem, traversal gap is leveraged to reduce the area of overlapped region, as shown in Figure 3. The black dots represent the upper-left pixel in each 4 × 4 block. The length between the centroids of two successively traversed windows is the traversal gap that depends on the size of the filter.

BMS has higher successful attack rate than MCwN; however, it requires prior knowledge of the filters.

## 4. Experiments

### 4.1. Dataset and Palmprint Recognition Methods

The palmprint database PolyU [33] was used in the experiments, which contains 7752 grayscale palmprint images taken from 386 palms in two sessions. The database was divided into two parts: PalmBigDatabaseA and PalmBigDatabaseB. The images in PalmBigDatabaseA were used as the target images, which are all attacked in the experiments. The images in PalmBigDatabaseB were used as the initial images for the attack methods. The PalmBigDatabaseA contains the first 1000 images of 50 palms, which were used as the target images. As described in Section 3, a real palmprint image, which can be easily obtained, is used as the initial image. PalmBigDatabaseB contains the images of 286 palms, which are randomly selected as the initial images. The two databases do not share the common palm categories, so it is impossible that the initial image and the target image are from the identical palm.

The experiments are conducted on eight coding-based palmprint recognition algorithms: PalmCode [36], BOCV [37], Ordinance Code [38], FusionCode [39], CompCode [40], RLOC [41], DOC [42], and DRCC [43]. The false non-match rate (FNMR) and false match rate (FMR) were calculated. When FNMR = FMR, their value is defined as equal error rate (EER). Normalized Hamming distance (NHD) measures the dissimilarity between two palmprint templates. Table 2 shows the NHD at EER, FNMR = 0, and FMR = 0 on PalmBigDatabaseA. The NHDs in Table 2 are not used for accuracy comparison, but to determine the different NHD thresholds at which the attack performance should be tested.

### 4.2. Attack Performance

Although many reconstruction algorithms exist, as summarized in Section 2.1, most of them are unsuitable for the palmprint modality. Our attacks are compared with Galbally et al.’s attack [26]. Galbally et al.’s attack uses multiple real iris images as the initial population and uses the matching distance as the fitness function. After multiple iterations of the genetic algorithm, the reconstructed image can impersonate the target user and cheat the iris recognition system. Then reconstructed image is embedded in a high-quality iris image to improve the naturalness and visual quality of the final reconstructed image. The reasons for choosing Galbally et al.’s attack for comparison are as follows: First, Galbally et al.’s attack is designed on a coding-based iris recognition system, which is similar to the coding-based palmprint recognition systems and can be applied to the coding-based palmprint recognition systems. Second, Galbally et al.’s attack requires the same prerequisites as the attacks proposed in this paper; i.e., the constant matching distance of each iterative modification. Third, the reconstructed images generated by their method have a medium naturalness and visual quality.

To test [26], a palmprint image is randomly selected from PalmBigDatabase as the embedded image. For our attacks, an image is randomly selected from PalmbigDatabaseB as the initial input. Each palmprint image in PalmbigDatabaseA is a target image. We list the successful attack rates at FNMR = 0, EER, FMR = 0, the average number of matching, peak signal-to-noise ratio (PSNR), and structural similarity (SSIM). PSNR and SSIM are calculated between the reconstructed ROI image and the initial ROI image. The two methods proposed in this paper and the compared methods all use a high-quality real palmprint as an initial image, and the reconstructed image is iteratively modified on the basis of it. It can be considered that the real palmprint image has high naturalness, so the PSNR and SSIM values between the reconstructed image and initial image can be calculated. The lower the PSNR and SSIM values are, the larger the modification range is, and the more seriously the naturalness degrades. The results of the different coding-based palmprint recognition methods are shown in Table 3, Table 4, Table 5, Table 6, Table 7, Table 8, Table 9 and Table 10.

All the methods yielded an attack success rate of nearly 100%. The matching number refers to how many times the reconstructed image was modified; i.e., how many times the fake template was matched with the target template. Since all these methods are based on iterative evolution, the matching number, as an important dynamic characteristic, measures the convergence speed. In addition, our methods have advantaged the naturalness and visual quality. Since the initial image is a real palmprint image, it is possible to satisfy similarity at FNMR = 0 or even EER at the beginning, so infinite (Inf) PSNR is possible. The number of the initial images with Inf PSNR was recorded, and these images were excluded when calculating the PSNR.

In [26], 10 real palmprints are used as the initial individuals, so it is more probable to satisfy similarity at the beginning at FNMR = 0. However, the NHD is very large at FNMR = 0, so the threshold is commonly set at EER, and the results at FNMR = 0 are unimportant. The following discussions focus on the results at EER and FMR = 0. BMS has lower matching numbers, so its computational complexity is low. Both BMS and MCwN outperform [26] in terms of PSNR and SSIM; while BMS yields the best results of PSNR and SSIM. In [26], several palmprint images are required as the initial individuals, while our attacks need only one palmprint image as the initial input.

**Figure 4:**
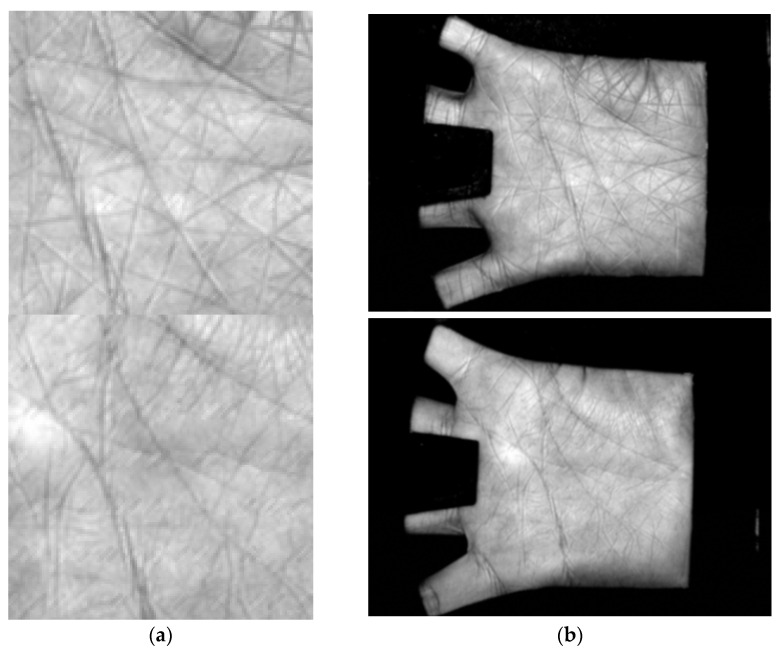
Reconstructed ROI and complete fake palmprint image: (**a**) the reconstructed ROI; and (**b**) the complete palmprint images with the embedding of the reconstructed ROI.

The reconstructed ROI image can be embedded into its original complete palmprint image to replace the original ROI region. Such a complete palmprint image, into which the reconstructed ROI is embedded, can be input into the system and pre-processed. Since the modifications in our attacks are slight, the difference between the reconstructed ROI image and its original version is weak. It is difficult to find the forgery appearance in the final complete palmprint image, as shown in Figure 4.

### 4.3. Ablation Experiment

**Figure 5:**
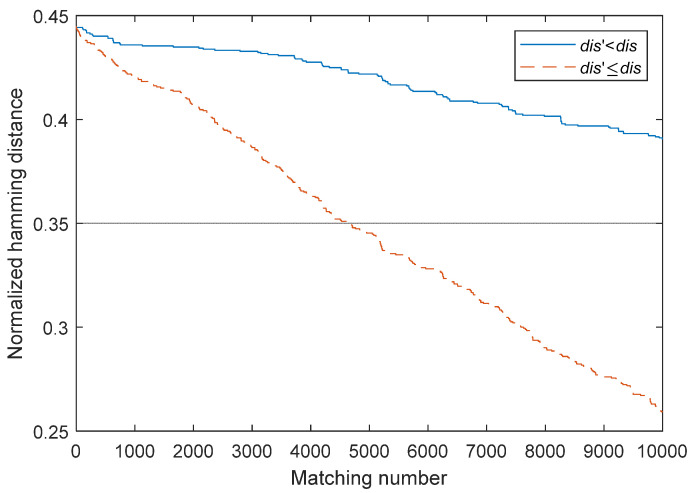
Two modification modes when distance is not changed.

Figure 5 compares the results of the conditions $dis^{\prime }\leq dis$ and $dis^{\prime }<dis$ of single-pixel modification on PalmCode [36]. The attack of $dis^{\prime }\leq dis$ is much better than that of $dis^{\prime }<dis$, because the modifications are conducted when $dis^{\prime }=dis$, which ensures the momentum is enough to keep persistently reducing the matching distance.

**Figure 6:**
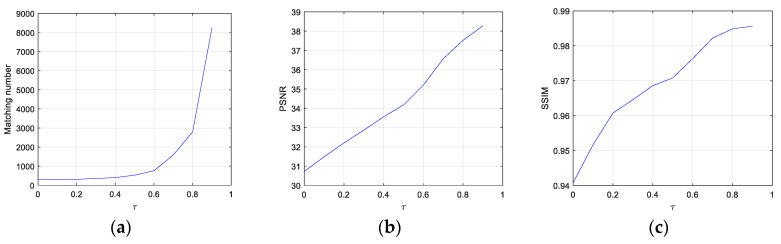
The effects of the threshold for BMS: (**a**) Matching number; (**b**) PSNR; (**c**) SSIM.

Figure 6 shows the effects of τ on matching numbers, PSNR, and SSIM. All the three values increase with the increment in τ. The increments in PSNR and SSIM are stable and durative, while the matching number increases slowly at first, but sharply so when τ > 0.6.

**Figure 7:**
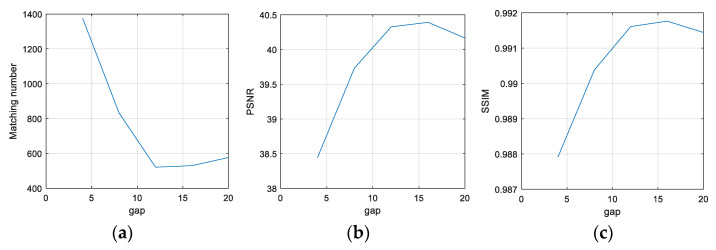
The effects of a traversal gap on batch modification: (**a**) Matching number; (**b**) PSNR; (**c**) SSIM.

Figure 7 shows the effects of the traversal gap on matching number, PSNR, and SSIM. The matching number decreases at first and then increases, while the trends in PSNR and SSIM are just the opposite. The results are best when the gap is 12.

## 5. Conclusions and Future Works

In this paper, in order to comprehensively evaluate biometric reconstruction attacks in secure identity management systems, more indicators are proposed, including similarity, naturalness, visual quality, and completeness. The existing reconstruction attacks cannot simultaneously satisfy the indicators. Two novel reinforced reconstruction attacks with reinforcement strategies are proposed for palmprints, which is a promising and representative biometric modality. Any palmprint image, which can be easily obtained, is used as the initial image, and the region of interest is iteratively modified to reduce the matching distance. Modification Constraint within Neighborhood (MCwN) and Batch Member Selection (BMS) are proposed, which have completeness and can maximize the naturalness and visual quality. In addition, BMS has a much smaller matching number and lower computational complexity. The two reinforced attacks can satisfy all the requirements, which cannot be simultaneously satisfied by the existing attacks. Our works show that unprotected biometric templates are vulnerable to reconstruction attacks. In the future, we will test the attack performance of the proposed methods on biometric systems with template protection. We will also try to design defense methods to resist reconstruction attacks in biometric systems.

## Figures and Tables

**Table 1:** Comparison of the reconstruction attacks in biometric systems (L, M, and H denote low, medium, and high, respectively).

Ref.	Year	Modality	Methodology	Naturalness	Visual Quality
[6]	2001	Fingerprint	The orientations were reconstructed from the singular points (core, delta) based on pole zero model. Some lines were drawn through the details, resulting in only a sketch of the fingerprints.	L	M
[7]	2004	Fingerprint	The minutiae image was reconstructed using HC.	L	L
[8]	2007	Fingerprint	The direction, category and ridge of the original fingerprint were extracted from the minutiae template.	L	M
[9]	2007	Fingerprint	Local detail model was used to initialize the image, and then Gabor filter was iteratively applied to the image formed by the detail parts.	M	M
[10]	2009	Fingerprint	The orientation field was used to reconstruct the continuous phase that was combined with spiral phase.	M	M
[11]	2011	Fingerprint	The phase image was reconstructed from fingerprint minutiae template, and then converted into a gray image.	M	H
[12]	2012	Fingerprint	A binary ridge pattern was generated, which has a similar ridge flow to that of the original fingerprint. The continuous phase was intuitively reconstructed by removing the spirals in the phase image estimated from the ridge pattern.	M	H
[13]	2015	Fingerprint	The prior knowledge of fingerprint ridge structure was coded through the direction patch and continuous phase patch dictionary. Then the direction field and ridge pattern were reconstructed.	M	H
[14]	2018	Fingerprint	Fingerprint images were reconstructed using cGNA and fingerprint minutiae templates.	M	H
[15]	2003	Face	A candidate image was slightly modified by an eigenface image, and the modifications improving the match score were kept.	L	L
[16]	2004	Face	Face images were reconstructed using HC.	L	L
[17]	2007	Face	Given the coordinates of the targeted subject in the affine space, the original template was reconstructed based on inverse affine transformation.	M	H
[18]	2009	Face	The HC based on Bayesian adaption was used to reconstruct face images.	M	H
[19]	2010	Face	According to the global distribution calculated on the user set, the local characteristics of the attacked client are adapted.	M	H
[20]	2012	Face	The HC based on uphill-simplex algorithm was used to reconstruct face images.	M	H
[21]	2013	Face	A simple reconstruction method was proposed based on RBF regression in face eigenspace.	M	H
[22]	2014	Face	Perceptual learning and HC were used to reconstruct real-valued features from the binary template.	M	M
[23]	2018	Face	A Neighbor Deconvolutional Neural Network (NbNet) was proposed to reconstruct face images from deep face templates.	M	M
[24]	2010	Iris	The initial template was divided into blocks of the same size. The pixels in blocks were modified by genetic algorithm.	M	M
[25]	2011	Iris	The texture image was generated from iris template and embedded into a real iris image.	M	M
[26]	2013	Iris	Genetic algorithm was used to reconstruct images from binary templates.	M	M
[27]	2020	Palmprint	Palmprint images were generated by Generative Adversarial Network (GAN) for false acceptance attack.	H	H

**Table 2:** Normalized Hamming distances at EER, FNMR = 0, and FMR = 0 on PalmBigDatabaseA.

	NHD (FNMR = 0)	NHD (EER)	NHD (FMR = 0)
PalmCode [36]	0.425	0.370	0.330
BOCV [37]	0.450	0.390	0.365
OrdinalCode [38]	0.440	0.340	0.285
FusionCode [39]	0.430	0.370	0.335
CompCode [40]	0.160	0.130	0.115
RLOC [41]	0.475	0.410	0.390
DOC [42]	0.465	0.420	0.400
DRCC [43]	0.445	0.390	0.360

**Table 3:** Attack performance of PalmCode [36].

PalmCode [36]	Matching Number (Mean) ↓	Matching Number (Std) ↓	PSNR (Inf) ↑	PSNR (Mean) ↑	PSNR (Std) ↓	SSIM (Mean) ↑	SSIM (Std) ↓
Galbally (FNMR = 0)	1.0	6.760	940	23.2	3.392	0.988	0.050
Galbally (EER = 0)	465.6	364.91	15	24.1	2.754	0.795	0.087
Galbally (FMR = 0)	1618.7	893.351	1	22.2	1.855	0.695	0.086
MCwN (FNMR = 0)	1108.3	1101.942	317	35.0	3.868	0.972	0.028
MCwN (EER)	6013.9	1791.945	4	28.6	1.635	0.866	0.045
MCwN (FMR = 0)	9866.2	2192.273	0	26.4	1.179	0.794	0.060
BMS (FNMR = 0)	86.3	82.409	293	48.3	5.133	0.999	0
BMS (EER)	577.1	246.770	4	40.3	1.658	0.992	0
BMS (FMR = 0)	1511.2	655.849	0	36.9	1.098	0.983	0

**Table 4:** Attack performance of BOCV [37].

BOCV [37]	Matching Number (Mean) ↓	Matching Number (Std) ↓	PSNR (Inf) ↑	PSNR (Mean) ↑	PSNR (Std) ↓	SSIM (Mean) ↑	SSIM (Std) ↓
Galbally (FNMR = 0)	0	0	0	36.1	1.574	0.973	0
Galbally (EER = 0)	1117.8	875.181	0	28.7	2.546	0.927	0.022
Galbally (FMR = 0)	3731.1	2743.178	0	27.9	1.894	0.913	0.020
MCwN (FNMR = 0)	248.5	587.795	727	40.2	5.835	0.995	0.010
MCwN (EER)	8865.1	2484.256	1	29.3	1.708	0.880	0.037
MCwN (FMR = 0)	13,549.7	3292.596	0	27.5	1.400	0.822	0.050
BMS (FNMR = 0)	23.5	42.123	389	54.9	7.598	0.999	0
BMS (EER)	1188.3	546.249	0	34.7	1.999	0.977	0.010
BMS (FMR = 0)	2261.9	903.541	0	32.5	1.058	0.964	0.010

**Table 5:** Attack performance of OrdinalCode [38].

OrdinalCode [38]	Matching Number (Mean) ↓	Matching Number (Std) ↓	PSNR (Inf) ↑	PSNR (Mean) ↑	PSNR (Std) ↓	SSIM (Mean) ↑	SSIM (Std) ↓
Galbally (FNMR = 0)	0	0	998	/	/	1	0
Galbally (EER = 0)	783.5	617.210	47	23.8	2.535	0.785	0.100
Galbally (FMR = 0)	5656.8	4221.575	0	20.6	1.392	0.579	0.094
MCwN (FNMR = 0)	67.7	360.238	944	36.5	4.875	0.998	0.010
MCwN (EER)	9577.8	3352.793	3	26.8	1.813	0.797	0.076
MCwN (FMR = 0)	20,139.2	6879.100	0	23.8	1.646	0.626	0.114
BMS (FNMR = 0)	6.8	15.533	944	41.8	4.020	1.000	0
BMS (EER)	742.8	1051.393	3	30.2	2.075	0.943	0.022
BMS (FMR = 0)	1956.0	2222.085	0	26.9	1.135	0.895	0.026

**Table 6:** Attack performance of FusionCode [39].

FusionCode [39]	Matching Number (Mean) ↓	Matching Number (Std) ↓	PSNR (Inf) ↑	PSNR (Mean) ↑	PSNR (Std) ↓	SSIM (Mean) ↑	SSIM (Std) ↓
Galbally (FNMR = 0)	0	0	997	15.2	0	1.000	0.010
Galbally (EER = 0)	789.0	912.764	30	23.7	2.539	0.776	0.096
Galbally (FMR = 0)	4544.6	3667.629	0	21.2	1.554	0.617	0.093
MCwN (FNMR = 0)	320.6	693.742	729	36.6	4.719	0.992	0.017
MCwN (EER)	6968.7	2152.851	4	28.4	1.788	0.856	0.050
MCwN (FMR = 0)	11660.5	2540.917	0	26.2	1.260	0.774	0.064
BMS (FNMR = 0)	25.4	48.533	647	52.8	6.235	1.000	0
BMS (EER)	952.4	692.114	3	39.5	2.185	0.991	0
BMS (FMR = 0)	2972.4	2503.948	0	36.6	1.162	0.984	0

**Table 7:** Attack performance of CompCode [40].

CompCode [40]	Matching Number (Mean) ↓	Matching Number (Std) ↓	PSNR (Inf) ↑	PSNR (Mean) ↑	PSNR (Std) ↓	SSIM (Mean) ↑	SSIM (Std) ↓
Gabally (FNMR = 0)	0	0	998	/	/	1	0
Gabally (EER = 0)	1535.4	1323.859	7	23.1	2.717	0.736	0.114
Gabally (FMR = 0)	7630.8	6221.137	0	20.7	1.492	0.586	0.100
MCwN (FNMR = 0)	5.2	41.978	976	45.8	6.306	1.000	0
MCwN (EER)	6600.8	1782.556	0	27.9	1.618	0.839	0.052
MCwN (FMR = 0)	11,016.4	2224.044	0	25.6	1.179	0.749	0.070
BMS (FNMR = 0)	4.4	3.255	787	54.6	3.308	1.000	0
BMS (EER)	394.6	173.592	0	34.4	1.613	0.978	0.010
BMS (FMR = 0)	1081.9	711.593	0	31.4	0.941	0.960	0.010

**Table 8:** Attack performance of RLOC [41].

RLOC [41]	Matching Number (Mean) ↓	Matching Number (Std) ↓	PSNR (Inf) ↑	PSNR (Mean) ↑	PSNR (Std) ↓	SSIM (Mean) ↑	SSIM (Std) ↓
Galbally (FNMR = 0)	0	0	998	/	/	1.000	0
Galbally (EER = 0)	1094.7	845.827	9	23.6	2.587	0.763	0.098
Galbally (FMR = 0)	2809.6	1691.399	0	22.0	1.861	0.676	0.093
MCwN (FNMR = 0)	16.0	99.884	959	41.4	4.549	1.000	0
MCwN (EER)	4514.2	1282.262	0	29.2	1.592	0.887	0.036
MCwN (FMR = 0)	6416.3	1386.419	0	27.6	1.156	0.843	0.041
BMS (FNMR = 0)	6.2	12.336	814	60.4	4.705	1.000	0
BMS (EER)	1628.5	605.921	0	39.1	1.735	0.988	0
BMS (FMR = 0)	2561.4	692.470	0	37.3	1.065	0.982	0

**Table 9:** Attack performance of DOC [42].

DOC [42]	Matching Number (Mean) ↓	Matching Number (Std) ↓	PSNR (Inf) ↑	PSNR (Mean) ↑	PSNR (Std) ↓	SSIM (Mean) ↑	SSIM (Std) ↓
Galbally (FNMR = 0)	0	0	998	/	/	1.000	0
Galbally (EER = 0)	973.0	831.115	12	23.7	2.628	0.767	0.097
Galbally (FMR = 0)	3595.0	2740.069	0	21.6	1.610	0.647	0.091
MCwN (FNMR = 0)	159.7	396.768	741	40.9	5.285	0.996	0.010
MCwN (EER)	6569.4	1802.938	0	30	1.850	0.896	0.033
MCwN (FMR = 0)	10,054.7	1934.229	0	28.2	1.316	0.851	0.040
BMS (FNMR = 0)	17.3	28.167	448	56.7	6.767	1.000	0
BMS (EER)	813.6	809.699	0	38.8	1.890	0.990	0
BMS (FMR = 0)	1839.0	1896.114	0	36.4	1.078	0.984	0

**Table 10:** Attack performance of DRCC [43].

DRCC [43]	Matching Number (Mean) ↓	Matching Number (Std) ↓	PSNR (Inf) ↑	PSNR (Mean) ↑	PSNR (Std) ↓	SSIM (Mean) ↑	SSIM (Std) ↓
Galbally (FNMR = 0)	0	0	998	/	/	1.000	0
Galbally (EER = 0)	815.2	892.207	21	23.4	2.608	0.766	0.097
Galbally (FMR = 0)	6313.7	4771.276	0	20.7	1.402	0.590	0.084
MCwN (FNMR = 0)	45.5	213.826	924	41.3	6.267	0.999	0
MCwN (EER)	6841.4	1982.581	1	29.5	1.691	0.884	0.038
MCwN (FMR = 0)	12,168.2	2467.356	0	27.2	1.221	0.810	0.050
BMS (FNMR = 0)	7.3	14.359	585	60.5	5.091	1.000	0
BMS (EER)	693.3	373.336	1	39.3	1.905	0.991	0
BMS (FMR = 0)	2164.1	1533.112	0	36.1	1.008	0.983	0

## Data Availability

Not applicable.
